# Chicago Medical Society

**Published:** 1883-06

**Authors:** 


### ARTICLE V.

### CHICAGO MEDICAL SOCIETY.

The meeting of April 16, 1883, with Dr. D. W. Graham in the chair, was the largest attended meeting since that of four years ago, when Dr. Cutter, of Boston, delivered his lecture on The Diagnosis of Syphilis by Examining the Blood with the Microscope. About 100 members and guests were present upon each occasion.

Drs. F. D. Porter and W. H. Wilson were elected to membership, and Dr. Robert Randolph, graduate of Bellevue, April 1879, proposed for membership by Drs. T. W. Miller and D. A. K. Steele.

Dr. Edmund Andrews first gave a brief and practical lecture on Litholopaxy, and exhibited a large calculus that he had crushed at one operation, in a patient's bladder, that P. M, by Bigelow's method, which was unattended by pain to the patient.

Dr. E. F. Ingals had prepared a very learned paper on the Treatment of Emphysema, which was read by Dr. W. L. Dorland.

Dr. Andrews moved, duly seconded, that discussion on Dr. Ingals' paper be postponed until the next meeting, as they had another scientific paper of considerable length to listen to this evening. The motion prevailed.

Dr. Wm. T. Belfield then proceeded to give a running commentary on Micro-Organisms in Disease, by exhibiting with a lantern and screen some thirty micro-photographs that were used to illustrate the recent Cartwright Lectures. The doctor consumed about an hour and a quarter in talking on this most interesting theme, during which the following named organisms were brought into view and dilated on :

1. Mole fungi, discovered in 1871, called ætomices bovis, although but 19 cases have been reported; found in man up to last August, in Europe.
2. Chromidia, or the flower part, which later develops to a seed.
3. Micrococci, as appears in septic poisoning as the rod bacillus ; as found in hay ; and the anthracis bacillus as found in animal blood.
4. Anthrax bacilli from kidney (rod-shaped).
5. Anthrax bacilli from liver.
6. Thread-growing bacilli in the blood.
7. Thread, still more magnified, with spores inside of threads.
8. A mass of foreign bacteria with threads and rods.
9. Bacilli from erysipelatous patient (supposedly so).
10. Section of heart-muscle from pyæmia (magnified 100 diameters) ; this represents a vessel plugged with micrococci.
11. Abscess from kidney from a case of ulcerous endocarditis.
12. Bacteria found in a malignant pustule on the hand.
13. Other forms of micrococci into fours, etc.
14. Section of kidney from a case of small-pox.
15. Piece of same kidney much more magnified.
16. Bacillus of typhoid-fever-spleen, liver and kidney.
17. Same case of typhoid ; more bacilli, more fully magnified.
18. Bacilli from pneumonia.
19. Bacilli from kidney in case of diphtheria of bladder.

20. Bacilli again taken from two forms of pyæmia.

21. Bacilli similar to that of anthrax, found in septicæmia.

22 and 23. Others similar, found in malignant œdema; found in spleen.

24. Bacilli, found in every cadaver 24 to 36 hours after death in warm weather.

25. Spirillum of recurrent fever or relapsing fever.

26. Tubercle bacilli of retro-peritoneal gland.

27. Section of the skin in a case of leprosy (bacilli rarely occur in this disease).

28. Filariæ sanguinis hominis (a worm).

29. The worm enlarged, found from 5 to 6 P. M. in a patient who had chyluria, to 5 to 6 A. M.; they are very active, 80 to 100 are found in motion in a drop of blood.

30. The trichina—also filariæ.

31. Filaria again, similar in structure to the trichina.

Dr. D. R. Brower moved that Dr. Belfield receive a vote of thanks for his interesting lecture that evening, which was an unanimous expression, followed by applause.

Regarding the delegates to the Illinois State Medical Society, upon motion of Dr. E. Ingals, the secretary was authorized to fill out alternates' certificates for vacancies occurring.

As the society is entitled to 42 delegates to the State Society, a motion of Dr. E. H. Thurston, that to facilitate matters, those desirous of attending should send their names to the secretary, was also carried, but few responded.

The following were voted delegates to that body:

Drs. P. M. Woodworth, D. R. Brower, J. A. Robison, Emma F. Gaston, Wm. P. Verity, E. H. Thurston; the list not yet being completed.

The following delegates to the American Medical Association were then nominated and elected:

Drs. Truman W. Miller, Arthur R. Reynolds, Simon Strausser, W. L. Dorland, Ephraim Ingals, Wm. T. Belfield, Philip Adolphus, A. B. Hosmer, Frank S. Johnson, T. W. Brophy, H. P. Newman, Christian Fenger, L. H. Montgomery, Chas. T. Parkes, F. H. Martin, Edwin Powell, D. W. Graham, E. F. Ingals, R. E. Starkweather, W. H. Curtis, G. C. Paoli.

Dr. L. H. Montgomery moved that a committee be appointed to confer with the passenger departments of the different railroads leading to Peoria and Cleveland relative to rates for delegates, and it was so ordered. Upon motion of Dr. N. S. Davis, the President and Secretary were appointed the committee.

Dr. E. Ingals, chairman of the committee appointed at the annual meeting to consider the suggestions embodied in the report of the secretary, presented the following:

TO THE CHICAGO MEDICAL SOCIETY:

Your committee to whom was referred the recommendations of your secretary, contained in his annual report, beg leave to report as follows:

1*st.* The proposed amendment to the constitution, which was to add a second vice-president to the officers of the society, has already been adopted, and requires no comment.

2*nd.* It is desirable that our membership should embrace every physician of good standing in the city, and as all names are submitted to the scrutiny of the committee on membership, it cannot matter by whom they are recommended.

3*rd.* We would recommend that the society provide for the publication of a brief history of its existence, with its constitution, and present list of members, with the date and place of their graduation.

4*th.* We deem it unwise for the society to add an insurance company as an auxiliary to its scientific purposes.

5*th.* We would recommend that a committee be appointed to obtain a charter for the society under the statutes of the State. This would perhaps add something to its reputation and perpetuity, and enable it to hold property in its corporate capacity.

6*th.* We would advise that the surplus money in the treasury be invested at interest, to serve, with future accumulations, as a fund by which we may ultimately obtain and own a permanent home for the society, and perhaps for some other kindred institutions.

All of which is respectfully submitted.

E. INGALS,<br>

D. W. GRAHAM,<br>

R. G. BOGUE,<br>

*Committee.*

Discussion being in order, Dr. E. Andrews argued at some length upon that portion of the report regarding a home for the society, etc., and concluded by moving that it be accepted and placed on file.

Dr. N. S. Davis thought it best to defer any further discussion until a future meeting, for he thought, as a rule, incorporated medical societies were liable to fall through in a few years. He moved as an amendment to the above motion, that the report be laid on the table for the present. The amendment was accepted and carried.

Dr. R. E. Starkweather suggested that in the notices on the next postal card announcements it should be stated that the quorum of delegates to the State Society had not yet been filled. It was so ordered.

The society adjourned at a late hour.

L. H. M.

The following is a partial list of members present:

Graham, Bettman, B., Elliott, Bates, Curtis W. H., Gaston, Mercer, Gradle, Engert, Potter, MacArthur, Martin, Steele, Starkweather, Coly, Davis N. S., Cook A. H., Colburn, Reynolds A. R., Adolphus, Dorland, Ingals E., Lescher, Brophy, Chew, Bidwell, Powell, Best, Randall, Weller, Montgomery L. H., Andrews E., Tucker D. M., Brower, Paoli, Johnson F. S., Burt, Tilley, Purdy, Robison, Verity, Thurston, Strausser, Stowel, Hobbs, Merriam, Fenger, Ingals E. F., Tagert, Hosmer, Hayes, Miller T. W., Woodworth.

The following visitors were in attendance:

Herbert C. Jones, M.D., Cerro Gordo, Ill.; J. W. Wier, M.D., Oswego, Kan.; J. S. Turner, M.D., Atalissa, Iowa; J. G. Langguth, Chicago; I. J. Smith; Harlan, Iowa; J. F. Sharp, J. T. T. Stoddard, A. Leigh, Highland, Kansas; W. F. Wakeman, City; Wm. Hoskins, City; A. T. Steele, M.D., Ashmon, Ill.

---

PROF. W. E. QUINE has resigned the chair of Materia Medica in Chicago Medical College, and accepted the chair of Practice of Medicine in the College of Physicians and Surgeons.

